# Fast and Sensitive Determination of the Fungicide Carbendazim in Fruit Juices with an Immunosensor Based on White Light Reflectance Spectroscopy †

**DOI:** 10.3390/bios11050153

**Published:** 2021-05-13

**Authors:** Georgios Koukouvinos, Chrysoula-Evangelia Karachaliou, Ioannis Raptis, Panagiota Petrou, Evangelia Livaniou, Sotirios Kakabakos

**Affiliations:** 1 Immunoassay/Immunosensors Lab, Institute of Nuclear & Radiological Sciences & Technology, Energy & Safety, National Centre for Scientific Research “Demokritos”, P.O. Box 60037, 15310 Agia Paraskevi, Greece; geokoukoubinos@yahoo.gr (G.K.); ypetrou@rrp.demokritos.gr (P.P.); skakab@rrp.demokritos.gr (S.K.); 2 Immunopeptide Chemistry Lab, Institute of Nuclear & Radiological Sciences & Technology, Energy & Safety, National Centre for Scientific Research “Demokritos”, P.O. Box 60037, 15310 Agia Paraskevi, Greece; 3 Institute of Nanoscience and Nanotechnology, National Centre for Scientific Research “Demokritos”, P.O. Box 60037, 15310 Agia Paraskevi, Greece; i.raptis@inn.demokritos.gr; 4 ThetaMetrisis S.A., Polydefkous 14, 12243 Egaleo, Greece

**Keywords:** white light reflectance spectroscopy, real-time immunosensor, ELISA, pesticides, carbendazim, fruit juices

## Abstract

Carbendazim is a systemic benzimidazole-type fungicide with broad-spectrum activity against fungi that undermine food products safety and quality. Despite its effectiveness, carbendazim constitutes a major environmental pollutant, being hazardous to both humans and animals. Therefore, fast and reliable determination of carbendazim levels in water, soil, and food samples is of high importance for both food industry and public health. Herein, an optical biosensor based on white light reflectance spectroscopy (WLRS) for fast and sensitive determination of carbendazim in fruit juices is presented. The transducer is a Si/SiO_2_ chip functionalized with a benzimidazole conjugate, and determination is based on a competitive immunoassay format. Thus, for the assay, a mixture of an in-house developed rabbit polyclonal anti-carbendazim antibody with the standards or samples is pumped over the chip, followed by biotinylated secondary antibody and streptavidin. The WLRS platform allows for real-time monitoring of biomolecular interactions carried out onto the Si/SiO_2_ chip by transforming the shift in the reflected interference spectrum caused by the immunoreaction to effective biomolecular adlayer thickness. The sensor is able to detect 20 ng/mL of carbendazim in fruit juices with high accuracy and precision (intra- and inter-assay CVs ≤ 6.9% and ≤9.4%, respectively) in less than 30 min, applying a simple sample treatment that alleviates any “matrix-effect” on the assay results and a 60 min preincubation step for improving assay sensitivity. Excellent analytical characteristics and short analysis time along with its small size render the proposed WLRS immunosensor ideal for future on-the-spot determination of carbendazim in food and environmental samples.

## 1. Introduction

Fungal contamination causes significant damages to the crops for human consumption every year, resulting in poor yield, deficient food quality, and huge economic loss. To circumvent these problems, the use of fungicides has been intensified over the last decades [1]. Carbendazim (methyl 1H-benzimidazol-2-ylcarbamate) is a synthetic, systemic, broad-spectrum, benzimidazole-type fungicide used worldwide as pre- and post-harvest treatment to control fungi that compromise the quality of various food commodities such as vegetables, fruits, cereals, and seeds [2,3]. Despite the unquestionable benefits regarding crop yield, carbendazim is a major pollutant, which induces acute and chronic effects on humans and livestock. In this context, carbendazim has been documented to induce infertility, embryotoxicity, teratogenicity, hepatocellular dysfunction, endocrine-disrupting effects, disruption of hematological functions, and mutagenicity [2]. Additionally, the World Health Organization has classified carbendazim as a possible human carcinogen [4]. Due to its aforementioned severe toxicities and its persistence in food and the environment, carbendazim has been officially banned in most of the European Union countries, USA, and Australia. However, its production and use in various formulations are still permitted in some countries, such as UK, Portugal, India, China, and Brazil [5], raising a growing concern for its adverse effects on the health of both humans and animals. To protect consumers from food products contaminated with carbendazim, regulatory authorities have established maximum residue limits (MRLs) for this pesticide in several matrices (fresh fruits and vegetables, oil seeds, cereals, spices, etc.). For example, the carbendazim MRL in fruit juices is 200 ppb and the MRL sum of benzimidazole pesticides has been set at 500 ppb by European Union [6,7]. The presence of carbendazim in fruit juice products has raised many concerns due to their worldwide popularity and the fact that children are their primary consumers [8].

The protection of the public health from pesticide residues in food requires, in addition to relevant legislation, accurate analytical methodologies. Determination of carbendazim is routinely performed by instrumental analytical techniques, mainly high-performance liquid chromatography coupled to mass spectroscopy or ultraviolet spectroscopy [9,10]. Alternatively, immunochemical techniques have been developed and used, offering low-cost and short-time analyses, simple assay protocols, and minimum sample pretreatment, and high-throughput sample analysis capacity. In this context, classic enzyme-linked immunosorbent assays (ELISA) [11] and immunochromatographic strips [12] have been reported in the literature, while immunosensors have lately attracted much attention due to their simplicity, rapidity, portability, and potential for the point-of-need application [13].

**Scheme 1:**
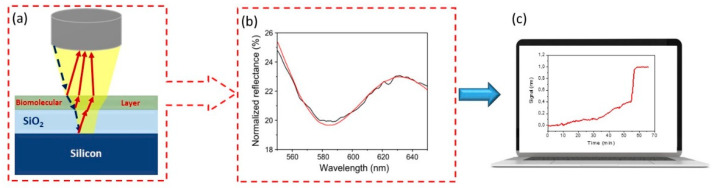
WLRS set-up and operation principle: (**a**) a schematic of the incident light beam (blue dotted line) reflection at the layers of different refractive index of a WLRS chip (red lines); (**b**) typical reflectance spectrum (black line) and its fitting by the sensor software (red line); (**c**) a depiction of the real-time signal monitoring resulting by the recorded spectrum processing by dedicated software.

In this work, a real-time immunosensor based on white light reflectance spectroscopy (WLRS) is employed for the accurate, fast, and sensitive determination of carbendazim in fruit juice samples with the potential for use at the point-of-need. The transducer is a Si chip with a 1-μm thick SiO_2_ layer on top and it is transformed to a versatile biosensing element through immobilization of a suitable recognition molecule. The optical set-up includes a white light source, a reflection probe consisting of a bundle of seven optical fibers; six at the periphery of the probe and one at the center, and a spectrometer. The six fibers at the periphery of the reflection probe guide the light from the source to the chip surface, while the seventh central fiber collects the light reflected by the chip and guides it to the spectrometer. As the light strikes the chip surface vertically, it is reflected by the silicon surface and by the transparent materials adlayers (silicon dioxide and biomolecular layer) of different refractive index. This way interference takes place at each wavelength resulting in an interference spectrum that is collected by the central fiber of the reflection probe. The increase of the biomolecular adlayer thickness due to binding reactions taking place onto the chip surface causes a shift of the interference spectrum. The software receives the interference spectrum from the embedded spectrometer and the effective biomolecular adlayer thickness (that is the signal of the WLRS sensor) is determined implementing the Levenberg–Marquart algorithm [14]. As this conversion is done by the software in real-time, the evolution of the effective biomolecular adlayer thickness in the course of the binding reactions occurring on a biochip surface could be monitored in real-time (Scheme 1). Thus, the WLRS biosensing platform allows for the label-free, real-time monitoring of biomolecular interactions carried out onto the Si/SiO_2_ chip with a detectable effective adlayer thickness <0.1 nm. A presentation of WLRS set-up and operation principle is presented in Scheme 1. The WLRS sensing principle has been successfully applied to the quantitative determination of both high and low molecular weight analytes into a plethora of matrices, after proper biofunctionalization of the sensing surface [14,15,16].

**Figure 1:**
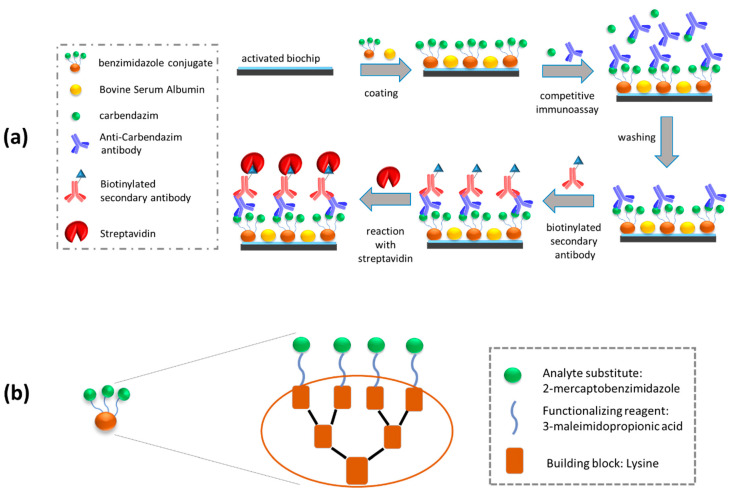
Schematic representation of (**a**) assay procedure for the detection of carbendazim with the WLRS sensor and (**b**) benzimidazole conjugate structure.

For carbendazim determination, a competitive immunoassay involving the delivery of a mixture of standards or fruit juice samples with a carbendazim-specific antibody to a benzimidazole conjugate-modified chip was conducted, as it is schematically depicted in Figure 1a. The primary immunoreaction, i.e., the competitive reaction between the immobilized onto the chip benzimidazole moieties and carbendazim in the standards or samples for the limited binding sites of the carbendazim-specific antibody [17], was followed by two signal enhancement steps. The first step included reaction with a biotinylated secondary antibody and the second one with streptavidin so as to further increase the thickness of the adlayer formed. The implementation of these two reactions aimed at the increase of the effective biomolecule adlayer thickness, i.e., the sensor signal, thus increasing the detection sensitivity. The benzimidazole conjugate [17] used for chip coating is also schematically presented in Figure 1b. Initially, a lysine-core peptidyl moiety was prepared using a fluorenylmethoxycarbonyl (Fmoc) solid phase peptide synthesis strategy previously described by us [18] with slight modifications. This moiety was then functionalized with 3-maleimidopropionic acid [19]. A benzimidazole derivative, 2-mercaptobenzimidazole, was then coupled to the 3-maleimidopropionic acid-functionalized peptidyl moiety through its thiol functional group. Notably, 2-mercaptobenzimidazole was used here because it is structurally similar to, but less toxic than, carbendazim. All assay parameters were optimized to achieve the highest possible detection sensitivity and the shortest assay duration. A simple sample preparation procedure was also developed to demonstrate the analysis of several commercially available fruit juice samples without any detectable “matrix-effect”. The accuracy of measurements with the proposed methodology was evaluated through recovery experiments using carbendazim-spiked samples prepared in commercially available fruit juices. Finally, the potential of regeneration and re-use of the biofunctionalized sensor chips was investigated, as a means to reduce the total cost of analysis. The novelty of the present work is mainly based on the combined use of an in-house prepared antibody for carbendazim recognition [17] and a benzimidazole derivative as a coating conjugate [17,18,19] in the WLRS immunoassay for carbendazim in different fruit juices.

## 2. Materials and Methods

### 2.1. Reagents and Materials

The rabbit polyclonal antibody for carbendazim (primary antibody) and the benzimidazole conjugate used for surface functionalization were in-house developed, as previously described [17]. Biotinylated goat anti-rabbit IgG (secondary antibody), streptavidin, streptavidin-horseradish peroxidase conjugate (streptavidin-HRP), 3,3′,5,5′-tetramethylbenzidine (TMB), carbendazim pestanal^®^, highly pure ethanol, acetone, isopropanol, and (3-aminopropyl)triethoxysilane (APTES) were from Sigma-Aldrich (St. Louis, MO, USA). Bovine serum albumin (BSA) was purchased from Acros Organics (Geel, Belgium). Polytetrafluoroethylene (PTFE) syringe filters of 0.45 μm pore size were the product of Membrane Solutions (Auburn, WA, USA). All other chemicals were purchased from Merck (Darmstadt, Germany). High-binding 96-well polystyrene microtiter plates (Code No. 3590) were purchased from Corning-Costar (Corning, NY, USA). Four-inch Si wafers (< 100 >) were purchased from Si-Mat Germany (Kaufering, Germany). These wafers were sequentially sonicated in acetone and isopropanol before a 1000-nm thick SiO_2_ layer was thermally oxidized on them at 1100 °C using the clean room facility at the Institute of Nanoscience and Nanotechnology of NCSR “Demokritos”. Then, the wafers were diced to chips with dimensions of 5 mm × 15 mm.

### 2.2. Preparation of Carbendazim Standard Solutions and Fruit Juice Samples

A 5 mg/mL carbendazim stock solution in absolute ethanol was prepared and stored at −20 °C. Standard solutions ranging from 20 ng/mL to 20 μg/mL prepared in 10 mM phosphate buffer, pH 7.4, containing 0.9% (*w/v*) NaCl, and 0.4% (*w/v*) BSA (assay buffer), were kept at −20 °C for up to 2 months. Fruit juices used in this study were purchased from local markets. In particular the following juices have been purchased: Amita orange and orange–lemon–carrot juice, and nine fruits Amita Motion juice from Coca-Cola 3E SA (Maroussi, Greece); orange, orange–apple–carrot, and nine fruits juice from Olympos Greek Dairies SA (Larissa, Greece); Eviva orange (short and long self-life) and lemon juice from Lidl Hellas (Sindos, Greece); Marata orange juice from Sklavenitis SA (Peristeri, Greece). All juices were separately filtered through a 0.45 μm-PTFE filter and the pH of the filtrate was adjusted to 7.4 ± 0.2 with 1 M NaOH solution.

### 2.3. Carbendazim-ELISA Assay

An indirect competitive ELISA was set up to conduct carbendazim detection. In brief, ELISA wells were incubated overnight in 100 μL of a 1 μg/mL benzimidazole conjugate in 0.05 M carbonate buffer, pH 9.2 (coating buffer). The plates were then washed three times with 10 mM phosphate buffered saline (PBS), pH 7.4, containing 0.05% (*v/v*) Tween-20 (PBS-T) using an ELISA plate washer (DIA Source), and incubated with 10 mM PBS, pH 7.4, containing 2% (*w/v*) BSA (200 μL/well), at room temperature for 1 h to block any remaining binding sites. After three washes with PBS-T, to each well were added 100 μL of 1:1 (*v/v*) mixtures of carbendazim standards (0.02–20 μg/mL in assay buffer) or samples and the anti-carbendazim antibody solution (2 μg/mL in assay buffer), which have been preincubated at room temperature for 1 h. After incubation at 37 °C for 90 min, the wells were washed three times with PBS-T, and incubated at 37 °C for 1 h with the biotinylated secondary antibody diluted 1:2000 in assay buffer (100 μL/well). The wells were then washed again with PBS-T and incubated at 37 °C for 45 min with a 750 ng/mL streptavidin-HRP solution in assay buffer (100 μL/well). The wells were washed again with PBS-T and incubated at room temperature for 20 min in 100 μL of chromogenic HRP substrate (TMB/H_2_O_2_). Finally, 50 μL of a 2 M aqueous sulfuric acid solution were added per well to terminate color development, and the absorbance of the wells at 450 nm (A_450_) was measured using a microtiter plate reader (Sirio S, Seak). An absorbance percentage was then calculated by dividing the absorbance of each standard (A_x_) to that of zero standard (A_0_). An absorbance percentage versus carbendazim concentration calibration plot was then constructed.

### 2.4. Evaluation of Primary Antibody—Specificity with the Carbendazim-ELISA Assay

The specificity of the primary antibody was evaluated through cross-reactivity studies with the pesticides carbaryl, imazalil, atrazine, and paraquat as follows. ELISA microwells were coated, blocked and washed as described in Section 2.3. Then, the wells were incubated at 37 °C for 90 min in 100 μL of a 1:1 (*v/v*) mixture (preincubated at room temperature for 1 h) of carbendazim standard solutions or standard solutions containing 0.02–5.0 μg/mL of each cross-reactant in assay buffer, and a 2 μg/mL anticarbendazim antibody solution in assay buffer. Microwells were washed, incubated with biotinylated secondary antibody, streptavidin-HRP, and TMB/H_2_O_2_ solution as described in Section 2.3. Finally, the absorbance was read at 450 nm, the calibration plots were constructed and the percent cross-reactivity (%CR) with each pesticide tested was determined according to the equation:

%CR = (IC_50_ carbedazim/IC_50_ cross-reactant pesticide) × 100

where IC_50_ carbedazim and IC_50_ cross-reactant represent the concentrations of carbendazim and cross-reactant in question, respectively, which provided 50% signal drop with respect to zero standard.

### 2.5. WLRS Instrumentation

WLRS instrumentation involves an FR-Pro tool operating in the 450–720 nm spectral range (ThetaMetrisis SA; Egaleo, Greece). The tool is equipped with a stabilized visible/near infrared light source and a high-performance miniaturized spectrometer tuned to provide very high optical resolution in the dedicated spectral range. The reflection probe delivers the incident light emitted by the source to the biofunctionalized chip surface through six fibers (diameter 400 μm) positioned to its periphery and collects the reflected light directing it to the spectrometer through a seventh fiber (diameter 400 μm) positioned at the center of the probe. The sensing surface consists of a transparent SiO_2_ film over a Si reflecting substrate covered by a custom-designed microfluidic cell (Jobst Technologies GmbH; Freiburg, Germany) providing the fluidic connections to the solutions. The FR-Pro tool was accompanied by a dedicated software that evaluates the initial thickness of the SiO_2_/protein adlayer and transforms in real-time the spectral shift due to the binding reactions in effective thickness of biomolecular adlayer expressed in nm. In more detail, a reference [*R*(*λ*)) and a dark spectrum (*D*(*λ*)] were acquired prior to the continuous recording of the reflectance spectrum [*S*(*λ*)], and the absolute reflectance spectrum is calculated by Equation (1):

(1) $$R\left(\lambda \right)=\frac{S\left(\lambda \right)-D\left(\lambda \right)}{R\left(\lambda \right)-D\left(\lambda \right)}$$

The normalized spectrum is then processed applying Levenberg–Marquart algorithm to calculate the thickness of the biomolecular adlayer, *d*_1_, from the shift in the interference spectrum wavelength, *δλ*, according to Equation (2):

(2) $$\delta \lambda =r_{1}\frac{1-r_{2}{}^{2}}{\left(r_{1}+r_{2})(1+r_{1}r_{2}\right)}\frac{n_{1}d_{1}}{n_{2}d_{2}}\lambda_{0m}$$

where, *r*_1_ and *r*_2_, and *n*_1_ and *n*_2_ are the Fresnel coefficients and refractive indices of the biomolecular and the silicon dioxide layer, respectively, *d*_1_ and *d*_2_ are the thickness of the two layers, and *λ* the wavelength.

### 2.6. Biochip Preparation and Biosensor Assay Performance

Chips were first cleaned and hydrophilized by O_2_ plasma treatment (10 mTorr) for 30 s in a reactive ion etcher. Then, they were immersed in a 2% (*v/v*) aqueous APTES solution for 20 min, gently washed with distilled water, dried under a nitrogen (N_2_) stream and cured by heating at 120 °C for 20 min. Chips were kept in a desiccator at room temperature for at least 48 h prior to use. For chip biofunctionalization, the benzimidazole conjugate (500 μg/mL, in coating buffer) was deposited on the chips and incubated at room temperature overnight. The following day, chips were rinsed with 10 mM PBS, pH 7.4 (washing buffer), blocked through immersion in 10 mM PBS, pH 7.4, containing 2% (*w/v*) BSA, for 3 h, rinsed with washing buffer and distilled water, dried with N_2_, and used for the assay (Figure 1). Prior to assay, each biofunctionalized chip was assembled with the fluidic module, placed on the docking station and equilibrated with assay buffer to acquire a stable baseline. For the assay, 1:1 (*v/v*) mixtures of standards (0.02–20 μg/mL in assay buffer) or samples with the rabbit anticarbendazim antibody (2 μg/mL in assay buffer), preincubated for 60 min, were passed over the chip for 18 min, followed by a 1:200 dilution of biotinylated anti-rabbit IgG antibody solution for 7 min, and a 10 μg/mL streptavidin solution in assay buffer for 3 min. The flow rate throughout the assay was 50 μL/min. Finally, the biochip was regenerated by running a 0.1 M glycine-HCl buffer, pH 2.5, for 3 min, followed by re-equilibration with assay buffer. The calibration plot was constructed by plotting the effective thickness of the built-up biomolecular adlayer (signal) corresponding to different standards, S_x_, expressed as percentage of the zero standard signal, S_0_ (maximum signal), against the carbendazim concentration in the standard solutions.

## 3. Results and Discussion

### 3.1. ELISA Assay for Carbendazim

Prior to the development of the carbendazim WLRS immunosensor assay, an ELISA in microtiter plates was set up to evaluate the basic immunoreagents used on the sensor platform, e.g., specificity of the anti-carbendazim antibody, as well as to determine optimal conditions for each assay step, e.g., optimum assay buffer. Moreover, the results obtained from the analysis of fruit juice samples with the ELISA assay were compared with those of the WLRS immunosensor.

#### 3.1.1. Assay Optimization

For the development of the competitive indirect ELISA for determination of carbendazim, a benzimidazole conjugate was used as a solid-phase reagent in combination with a rabbit polyclonal anticarbendazim antibody, both developed in-house as previously described [17]. The optimum concentration of the conjugate for coating was determined by preliminary titration experiments using mixtures of the anti-carbendazim polyclonal antibody with carbendazim standards prepared in assay buffer. As shown in Figure S1a, zero standard signal values in the range 1.0–1.5 (which are considered optimum for an ELISA) were received for the following combinations of conjugate/antibody concentrations 0.5/4.0, 1.0/2.0, and 2.5/1.0 in μg/mL. These combinations were tested further regarding not only the zero standard signal but also the signal received in presence of 200 ng/mL carbendazim. From Figure S1b, where the percent absorbance values are presented, it can be concluded that the combination that provided the highest percent signal drop in presence of carbendazim was 1.0 μg/mL benzimidazole conjugate and 2.0 μg/mL anticarbendazim antibody. Thus, this combination was adopted in the final ELISA protocol. Different assay buffers were then tested including a 10 mM PBS buffer, pH 6.5, 10 mM PBS, pH 7.4, 50 mM Tris-HCl buffer, pH 7.8, and 50 mM Tris-HCl buffer, pH 8.25, and the results obtained are shown in Scheme S1 (see Supplementary Material). All buffers contained 0.4% BSA. It was found that 10 mM PBS, pH 7.4, containing 0.4% (*w/v*) BSA, was the optimum buffer for the immunoreaction between the primary antibody and the antigen since it provided the highest signals for zero standard and detection sensitivity compared to the other buffers tested. The implementation of a preincubation step of the antibody with the standards prior to addition onto the biofunctionalized wells was investigated as a means to improve assay detection sensitivity. As shown in Figure S3, the assay sensitivity, expressed by the slope of the linear segment covering the two standards of the lowest concentration in the calibration plot, ranged from −0.19 [dS/(ng/mL)] for 0 min, −0.26 [dS/(ng/mL)] for 30 min, −0.42 [dS/(ng/mL)] for 60 min, and −0.38 [dS/(ng/mL)] for 120 min. These results indicate considerably improved assay sensitivity when a preincubation up to 60 min was used. However, longer preincubation did not result in any additional improvement. Thus, 60-min pre-incubation was adopted in the final protocol. Under optimal conditions, the detection limit of the assay (LoD, calculated as the carbendazim concentration corresponding to percent signal value equal to 100-3SD of 16 measurements of zero standard) was 20 ng/mL. The quantitation limit (LoQ) was calculated as the carbendazim concentration for which the mean absorbance value + 3SD is equal or lower than the mean zero standard value—3SD (LoD), and was 50 ng/mL. The dynamic range of the assay was from 50 ng/mL to 2 μg/mL.

The ELISA assay presented here differs from that previously described [17] in the configuration followed. More specifically, a biotinylated secondary antibody along with streptavidin-HRP have been used, instead of an HRP-labelled secondary antibody, while TMB (instead of 2,2′-azino-bis(3-ethylbenzothiazoline-6-sulfonic acid) diammonium salt—ABTS) has been employed as a chromogen (Scheme S1). Moreover, a preincubation step was adopted so as to further increase the detection sensitivity.

#### 3.1.2. Evaluation of the Anti-Carbendazim Antibody Specificity

**Figure 2:**
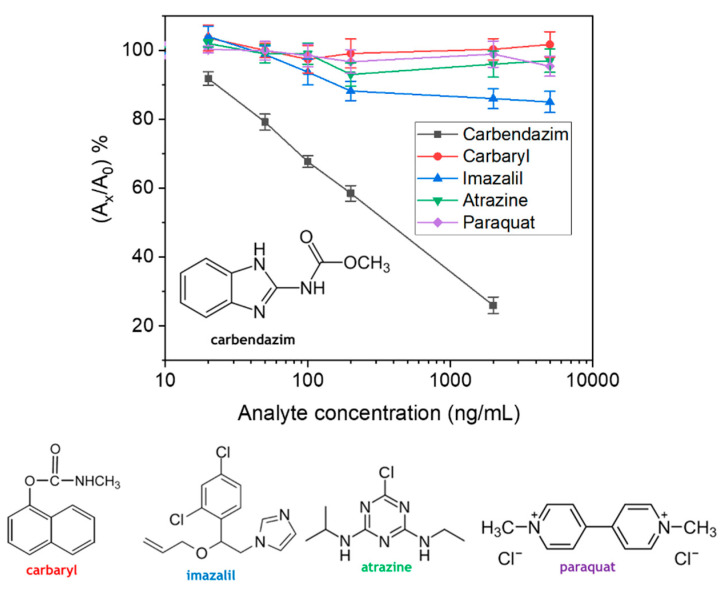
Calibration plots obtained with carbendazim (black squares), carbaryl (red circles), imazalil (blue up triangle), atrazine (green down triangle), and paraquat (purple rhombus) standards with concentrations ranging from 0 to 5000 ng/mL. All standards were preincubated for 60 min with the anti-carbendazim antibody prior to addition in the microwells. Each point is the mean value of 3 replicates ± SD.

The cross-reactivity of the anti-carbendazim antibody with four commonly reported pesticides in fruit juices [20,21,22], including carbaryl, imazalil, atrazine, and paraquat was tested. In Figure 2, the calibration plots obtained for each one of the four pesticides are provided. As a rule, compounds that do not provide at least 50% decrease in signal under the above described conditions, are not considered as cross-reacting materials. Thus, as shown, no cross-reactivity with any pesticide tested could be detected.

### 3.2. WLRS Assay

#### 3.2.1. Assay Optimization

The transfer of the carbendazim assay to the WLRS platform was based on results obtained in ELISA-optimization experiments, e.g., in terms of the composition of the assay buffer and the duration of the preincubation step. However, there were some parameters that also needed optimization. Due to the competitive nature of the assay, the two most crucial parameters to optimize were the concentration of benzimidazole conjugate used for coating and the concentration of anti-carbendazim antibody, as their combination determines the highest signal (zero standard signal) and the assay sensitivity. Hence, different concentrations of the benzimidazole conjugate (100–1000 μg/mL) in combination with different concentrations of the anti-carbendazim antibody (0.5–5.0 μg/mL) were tested. As shown in Figure S4, for all antibody concentrations tested, zero standard signal values increased as the concentration of benzimidazole-conjugate used for coating inreased and maximum signal plateau values were obtained for concentrations ≥500 μg/mL. The increase in the signal observed as the concentration of both benzimidazole-conjugate and anti-carbendazim antibody increases, depicts the increase in the biomolecular adlayer thickness due to the antigen–antibody reaction taking place onto the chip surface. Regarding the antibody concentration, it was found that the signal increased almost linearly up to an antibody concentration of 2 μg/mL, whereas higher antibody concentrations provided only marginal signal (10%) increase. In addition, as it can be concluded from Figure S5, the assay sensitivity deteriorated by approximately 50% when the anti-carbendazim antibody concentration increased from 2 to 4 μg/mL. Thus, a 2 μg/mL anti-carbendazim antibody concentration was adopted in the final assay protocol.

**Figure 3:**
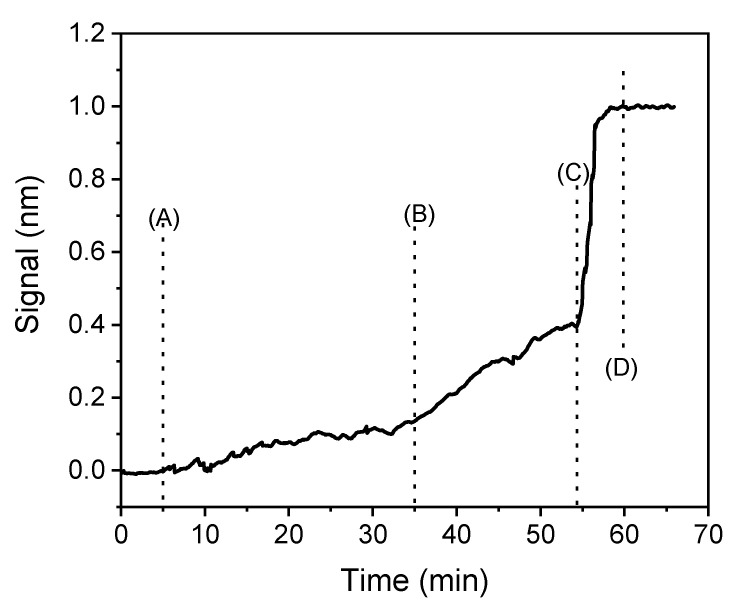
Real time response obtained from a biochip functionalized with 500 μg/mL of benzimidazole conjugate upon running over the chip: assay buffer (from start to point A); a 1:1 (*v/v*) mixture of zero standard with a 2 μg/mL rabbit anti-carbendazim antibody solution (A–B); a 1:200 diluted solution of a biotinylated secondary antibody (B–C); a 10 μg/mL streptavidin solution (C–D).

Another parameter to optimize was the duration of the whole assay, in order to achieve a fast and reliable assay. As shown in Figure 3, the primary immunoreaction reached a signal plateau after 30 min, the secondary immunoreaction in 20 min, and the reaction with streptavidin in 3 min. The real-time sensor response showed that the reaction with biotinylated secondary antibody and the subsequent reaction with streptavidin resulted in considerable signal enhancement. In particular, the reaction with the secondary antibody increased by 2.5-fold the signal obtained from the primary immunoreaction, whereas the reaction with streptavidin further increased the signal by approximately 3-times, leading to an overall 7.5-fold increase in the signal of the primary immunoreaction. The signal increase observed in the two reactions that follow the primary immunoreaction is ascribed to the fact that more than one biotinylated secondary antibody molecules bind per immunosorbed primary antibody, since the secondary antibody is a polyclonal one and has been raised against the whole anti-rabbit IgG molecule and it is expected, therefore, to bind to several epitopes of the primary antibody molecule. Similarly, the secondary antibody has several biotin-moieties and thus, more than one streptavidin molecule could bind per biotinylated secondary antibody molecule, providing considerable signal enhancement. Due to this accumulation of multiple molecules per immunosorbed primary antibody molecule, the effective biomolecular adlayer thickness, and consequently the WLRS sensor signal, is considerably increased.

**Figure 4:**
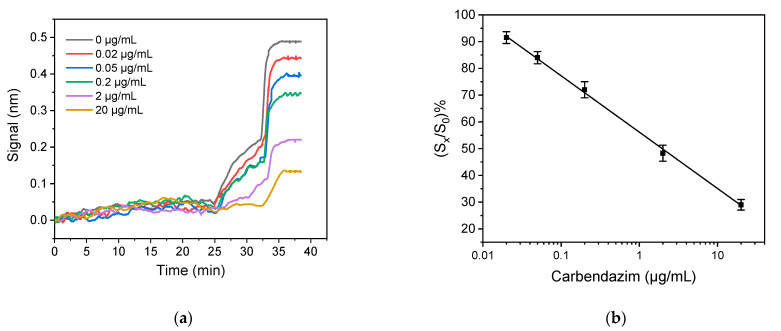
(**a**) Real-time responses obtained from biochips functionalized with 500 μg/mL benzimidazole conjugate upon running sequentially: assay buffer; a 1:1 *v/v* mixture of carbendazim standards (0–20,000 ng/mL) prepared in assay buffer with a 2 μg/mL anti-carbendazim antibody solution in the same buffer; a 1:200 diluted solution of biotinylated secondary antibody in assay buffer; a 10 μg/mL streptavidin solution. (**b**) Typical calibration plot for carbendazim. Each point represents the mean value of 4 runs ± SD.

The signal increase achieved by the implementation of the two signal enhancement steps allowed the reduction of the whole assay time. More specifically, the duration of the primary immunoreaction was set at 18 min, the reaction with the secondary antibody at 7 min, and that with streptavidin at 3 min, resulting in a total assay time of 28 min. This reduction in assay time reduced also the maximum signal obtained by 50%, however, the signal received with the shorter assay protocol was adequate for the performance of the assay. This was confirmed by the good discrimination of the real-time sensor responses obtained for carbendazim standards with concentrations ranging from 0 to 20 μg/mL as shown in Figure 4a. In addition, Figure 4b depicts the relevant calibration plot.

The LoD of the proposed immunosensor was evaluated as described in Section 3.1.1 for the respective ELISA assay and was found to be 20 ng/mL. The assay dynamic range was 50 ng/mL–20 μg/mL.

#### 3.2.2. Optimization of Sample Preparation Procedure

**Figure 5:**
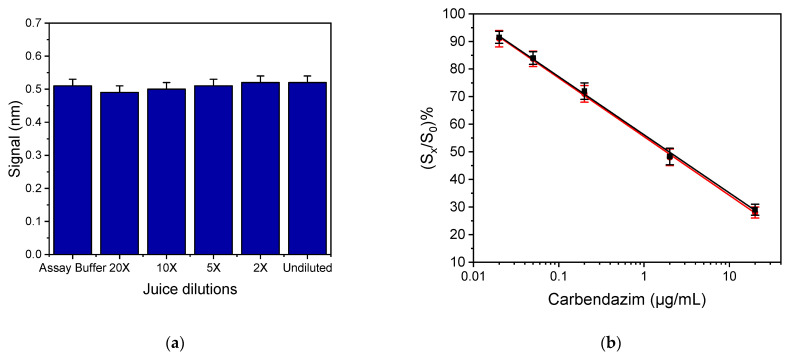
(**a**) Zero standard signal corresponding to assay buffer and filtered and pH-adjusted juice, undiluted and diluted 2× to 20× with assay buffer. Each bar represents the mean value of 3 independent measurements ± SD. (**b**) Typical calibration plots obtained with carbendazim standards prepared in assay buffer (black squares) and treated orange juice (red circles). Each point represents the mean value of 4 runs ± SD.

The developed immunosensor was applied to carbendazim detection in fruit juices. The acidic pH and the pulp in fruit juices are reportedly impacting the performance of immunoassays by affecting the antibody–antigen binding, resulting in the so-called “matrix-effect” [23,24]. Thus, it is necessary to perform a sample preparation procedure that minimises interferences before analysing fruit juice samples. In this respect, filtration through a 0.45 μm PTFE membrane syringe filter was performed to remove the pulp, and then the pH of the filtrate was adjusted to 7.4 ± 0.2 with addition of 1 M NaOH solution (without significantly changing the sample volume). In addition, to investigate the possible matrix effect of fruit juice samples to assay performance, the signal obtained by undiluted and 2- to 20-fold diluted samples was compared to that of zero standard signal in buffer. As shown in Figure 5a for an orange juice sample, the undiluted sample, which has been used after filtration and pH neutralization, provided the same signal with the assay buffer indicating that there was no need for additional sample dilution with assay buffer in order to alleviate the matrix effect. This is supported by the fact that the undiluted sample provided statistically the same zero standard values with orange juice diluted 2–20 times with assay buffer. Similar results were obtained using juices from other fruits, e.g., lemon, and from juices made from a combination of different fruits. Thus, all juices involved in the study were analyzed without dilution after filtration and pH adjustment. Further verification that there was not any matrix-effect in the assay performance was provided by the fact that the carbendazim calibration plots obtained in assay buffer and a suitably treated, commercial orange juice were superimposed, as presented in Figure 5b.

#### 3.2.3. Accuracy and Precision of the Developed ELISA and Sensor Assays

The accuracy of the measurements performed with the developed immunosensor was evaluated through recovery experiments using three commercially available juices prepared from different fruits. To this end, three fruit juices previously analyzed with the carbendazim ELISA and found not to contain any detectable carbendazim were fortified with concentrations of the pesticide that corresponded to three concentration levels, i.e., 100, 500, and 1000 ng/mL. The fortified samples were analyzed in triplicates both with the developed biosensor and the ELISA assay, prior to and after the addition of carbendazim. The results obtained from the analysis of the spiked samples are shown in Table 1, while no carbendazim could be detected in any of the samples prior to the addition of the pesticide. The percent recovery was calculated as the percent ratio of the carbendazim concentration determined in the spiked samples to that expected based on the amount spiked. The recovery values determined with the two methods are presented in Table 1. As shown, the recovery values obtained with the ELISA and the biosensor assays ranged from 90 to 110% and 89 to 110%, respectively, demonstrating the high accuracy of the assays developed. Furthermore, there was a very good agreement of the values determined with the immunosensor to those determined for the same samples with the ELISA. A paired *t*-test confirmed that there was not statistically significant difference between the results of the two methods (*p* < 0.05).

#### 3.2.4. Analysis of Commercially Available Fruit Juices

A survey on carbendazim residues in commercial packages of fruit juices was performed. Ten different juices of four different brands made from orange, lemon, or combination of different fruits were purchased from local supermarkets and analyzed by the WLRS sensor. The results are presented in Table 2. None of the products tested contained detectable amounts of carbendazim. This finding was confirmed by analysing the same samples with the carbendazim ELISA.

#### 3.2.5. Regeneration of Biochips

**Figure 6:**
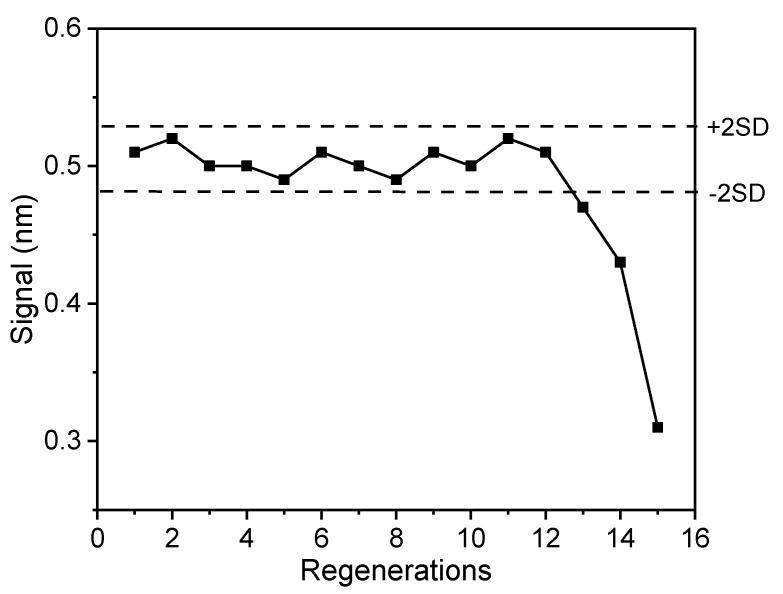
Signal responses obtained from a single biochip for 15 regeneration/assay cycles. Dashed lines represent the mean value ± 2SD limits.

The stability of the developed immunosensor response to sequential assay/regeneration cycles was also determined as a means to exploit the use of a single biochip for analysis of several samples thus reducing the analysis cost. A low-pH buffer is often used in affinity-based biosensor assays for surface regeneration purposes, to quantitatively remove the antibody from the coating analyte, without affecting the coating analyte structure. The regeneration was achieved by running a 0.1 M glycine-HCl buffer, pH 2.5, for 3 min after completion of the assay. Figure 6 shows the zero standard responses obtained from a single biochip in 15 assay/regeneration cycles performed over a period of three days. As shown, for up to 12 assay/regeneration cycles, all values consistently fell within the mean value ± 2SD range, which demonstrates the potential reuse of biosensor.

### 3.3. Comparison with Other Biosensors

In the last decade carbendazim has received particular attention from the research community and many efforts from research groups all over the world have been carried out towards the development of biosensors for carbendazim determination in various food commodities. The majority of the reported carbendazim biosensors are electrochemical [25,26,27,28,29,30,31]. Only few optical sensors for carbendazim detection have been reported in the literature. These include a sensor based on surface-enhanced Raman scattering technology, which involves cyclodextrin inclusion complexes on gold nanorods as recognition element [32], a sensor based on luminescence resonance energy transfer from aptamer-labeled upconversion nanoparticles to manganese dioxide nanosheets that act as an acceptor [33], and a surface plasmon resonance-based immunosensor [34]. The immunosensor proposed herein is the first optical sensor based on white light reflectance spectroscopy, dedicated to the determination of carbendazim in foodstuff.

With respect to actual analysis time of 28 min, our sensor is considered among the fastest sensors reported for carbendazim detection in foodstuff generally; even if the separate, 60-min preincubation step is taken into account, the proposed sensor can be still considered as an analytical tool capable of determining carbendazim very quickly. In terms of analytical sensitivity, the LoD of the developed method (20 ng/mL) is well below the current European Union regulatory limit of 200 ng/mL for carbendazim in fruit juices. It is noteworthy that in our case, as in the aforementioned SPR sensor [34], a signal enhancement step was introduced after the primary immunoreaction in order to generate a measurable response while keeping the analysis time as short as possible. Overall, the developed sensor could be regarded as a very fast and sensitive real-time biosensing platform for the detection of carbendazim, which could be considered as label-free, since it is not based on any typical label, such as a fluorophore or an enzyme, for the development of an optical signal. The sensor is accompanied by a rather simple sample preparation protocol that can be easily reproduced at the point-of-need as it does not require any special equipment. In addition, the sample preparation procedure could be applied to juices prepared from different fruits without any noticeable effect in the immunosensor performance by the difference in the sample matrix, which is very important for future on-site analysis of different juices.

## 4. Conclusions

In conclusion, a WLRS-based biosensing platform for the real-time immunochemical determination of carbendazim in fruit juices was successfully developed. The proposed sensor allowed for the sensitive and fast quantification of carbendazim levels down to 20 ng/mL within less than 30 min. Moreover, the fact that a single chip functionalized with the benzimidazole conjugate could be regenerated and reused for at least 12 times without any effect onto the signal received provides a further advantage. In summary, excellent analytical characteristics and short analysis time combined with the small size of the analytical device render the proposed WLRS biosensor ideal for future on-the-spot determination of carbendazim in food and environmental samples.

## Figures and Tables

**Table 1:** Recovery values of carbendazim in commercial fruit juices spiked with the indicated concentrations of the pesticide.

Juice Sample	Spiked Level (ng/mL)	Determined Concentration (ng/mL)		% Recovery	
		WLRS	ELISA	WLRS	ELISA
Orange	1000	1052 ± 53	1097 ± 41	105	110
	500	522 ± 32	540 ± 22	104	108
	100	98 ± 4	106 ± 3	98.0	106
Orange–apple–carrot	1000	973 ± 57	1010 ± 37	97.3	101
	500	547 ± 25	451 ± 26	109	90.2
	100	89 ± 25	91 ± 4	89.0	91.0
Lemon	1000	951 ± 61	896 ± 32	95.1	89.6
	500	487 ± 28	496 ± 18	97.4	99.2
	100	110 ± 7	113 ± 6	110	113

**Table 2:** Carbendazim residues in commercially available fruit juices. In the parentheses the trade name and storage conditions recommended by the manufacturers are provided.

Sample Number/Name (Storage)	Amount Detected (ng/mL)
1/ Orange juice (Amita, RT)	<LoD
2/ Orange juice (Eviva, RT)	<LoD
3/ Orange juice (Marata, RT)	<LoD
4/ Orange-lemon-carrot (Amita, RT)	<LoD
5/ 9 fruits Motion (Amita, RT)	<LoD
6/ Orange juice (Olympos, 4 °C)	<LoD
7/ Orange juice (Eviva, 4 °C)	<LoD
8/ Orange-apple-carrot (Olympos, 4 °C)	<LoD
9/ 9 Fruits (Olympos, 4 °C)	<LoD
10/ Lemon juice (Eviva, 4 °C)	<LoD

## Data Availability

The data presented in this study are available on request from the corresponding author. The data are not publicly available due to privacy issues.

## Supplementary Materials

The following are available online at https://www.mdpi.com/article/10.3390/bios11050153/s1, Scheme S1. TMB peroxidase substrate reaction. Figure S1. (a) Absorbance values at 450 nm received for zero carbendazim standard from wells coated with benzimidazole-conjugate concentration 0.5 (black squares), 1 (red circles), 2.5 (blue up triangles), or 5 μg/mL (green down triangles) when assayed with anti-carbendazim antibody concentrations ranging from 0.5 to 8 μg/mL. Each point is the mean value of four wells ± SD. (b) Percent absorbance values obtained for the zero carbendazim standard (orange bars) and a standard containing 200 ng/mL carbendazim (green bars) using different combinations of benzimidazole conjugate for well coating and anti-carbendazim antibody. Each point is the mean value of four wells ± SD. Figure S2. Absorbance values at 450 nm received for zero carbendazim standard (orange bars) and a standard containing 200 ng/mL carbendazim (green bars) using the following assay buffers: 10 mM PBS buffer, pH 6.5; 10 mM PBS, pH 7.4; 50 mM Tris-HCl buffer, pH 7.8; 50 mM Tris-HCl buffer, pH 8.25. All buffers contained 0.4% BSA. Each point is the mean value of four wells ± SD. Figure S3. Carbendazim calibration plots obtained without preincubation of carbendazim standards with the anti-carbendazim antibody solution (green down triangles) or with preincubation for 30 min (black squares), 60 min (red circles), and 120 min (blue up triangles). Each point is the mean value of four wells ± SD. Figure S4. Signals received for zero carbendazim standard from WLRS chips coated with benzimidazole conjugate concentrations ranging from 100 to 1000 μg/mL when assayed with anti-carbendazim antibody solutions of 0.5 (black squares), 1 (red circles), 2 (blue up triangles), or 4 μg/mL (green down triangles). Each point is the mean value of measurements obtained by three chips ± SD. Figure S5. Calibration plots obtained from chips coated with 500 μg/mL of benzimidazole conjugate and assayed with anti-carbendazim antibody solutions with concentration 2 (black squares) or 4 μg/mL (red circles). Each point is the mean value of three measurements ± SD.
