# Supplementary material for: Fast and Sensitive Determination of the Fungicide Carbendazim in Fruit Juices with an Immunosensor Based on White Light Reflectance Spectroscopy
Source: Biosensors (Basel). 2021 May 13;11(5):153. doi: 10.3390/bios11050153 (PMC8153324; doi:10.3390/bios11050153)
Supplement: Supplementary file 1 [file biosensors-11-00153-s001.zip › biosensors-1173726-SI.pdf]

Supplementary Material

a) TMB peroxidase substrate reaction

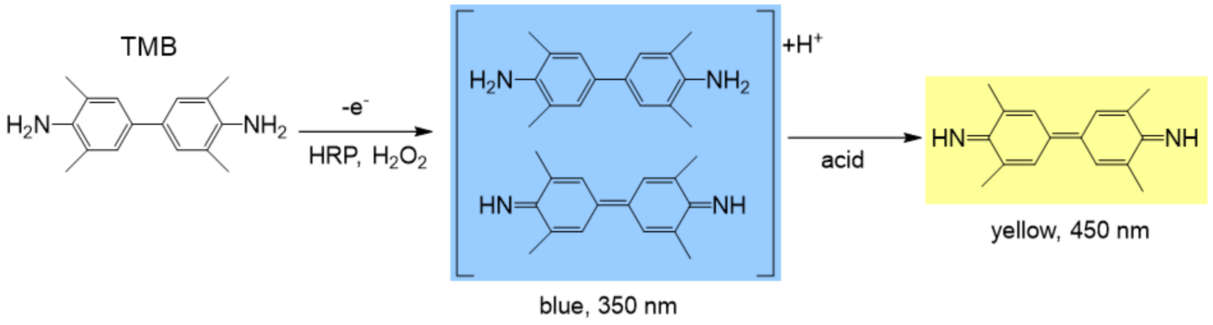

Scheme S1. TMB peroxidase substrate reaction.

b) Optimization of the carbendazim ELISA

i) Selection of benzimidazole conjugate and anti-carbendazim antibody concentrations

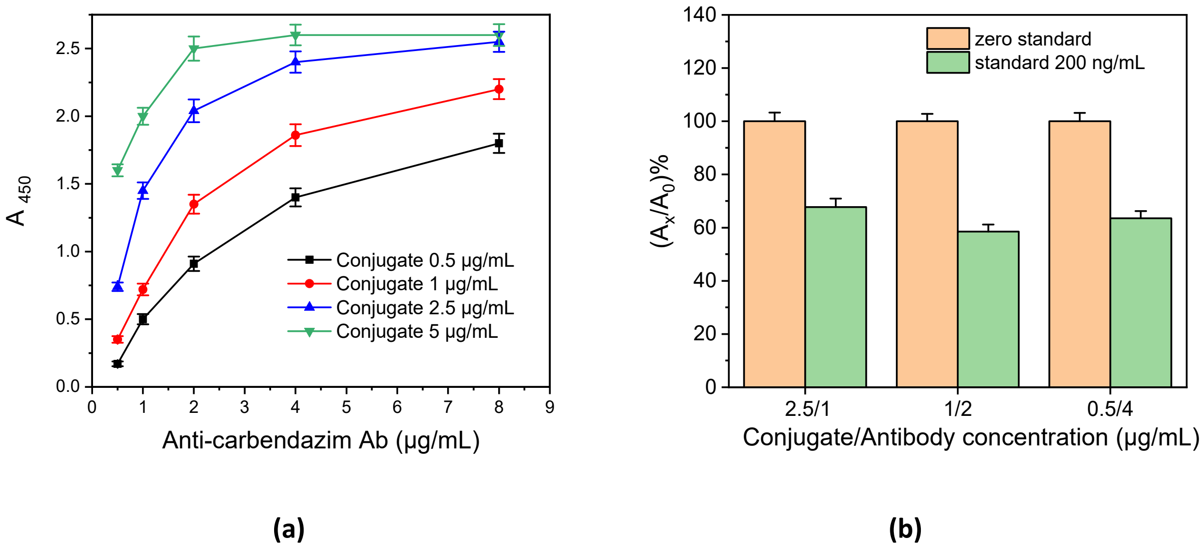

Figure S1. (**a**) Absorbance values at 450 nm received for zero carbendazim standard from wells coated with benzimidazole-conjugate concentration 0.5 (black squares), 1 (red circles), 2.5 (blue up triangles) or 5 μg/mL (green down triangles) when assayed with anti-carbendazim antibody concentrations ranging from 0.5 to 8 μg/mL. Each point is the mean value of four wells ± SD. (**b**) Percent absorbance values obtained for the zero carbendazim standard (orange bars) and a standard containing 200 ng/mL carbendazim (green bars) using different combinations of benzimidazole conjugate for well coating and anti-carbendazim antibody. Each point is the mean value of four wells ± SD.

ii) Selection of assay buffer

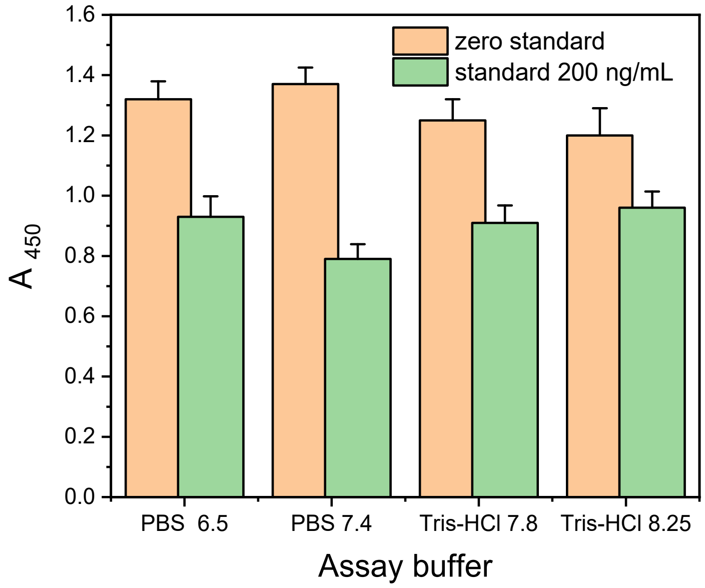

Figure S2. Absorbance values at 450 nm received for zero carbendazim standard (orange bars) and a standard containing 200 ng/mL carbendazim (green bars) using the following assay buffers: 10 mM PBS buffer, pH 6.5; 10 mM PBS, pH 7.4; 50 mM Tris-HCl buffer, pH 7.8; and 50 mM Tris-HCl buffer, pH 8.25. All buffers contained 0.4% BSA. Each point is the mean value of four wells ± SD.

iii) Effect of standard/antibody pre-incubation on the calibration curve

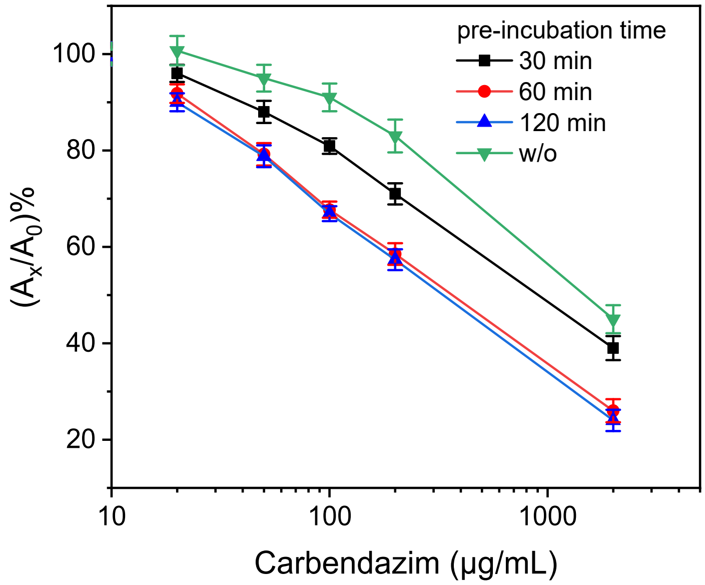

Figure S3. Carbendazim calibration plots obtained without pre-incubation of carbendazim standards with the anti-carbendazim antibody solution (green down triangles) or with pre-incubation for 30 min (black squares), 60 min (red circles), and 120 min (blue up triangles). Each point is the mean value of four wells ± SD.

c) <u>Optimization of the carbendazim WRLS-biosensor assay</u>

i) Selection of benzimidazole-conjugate concentration

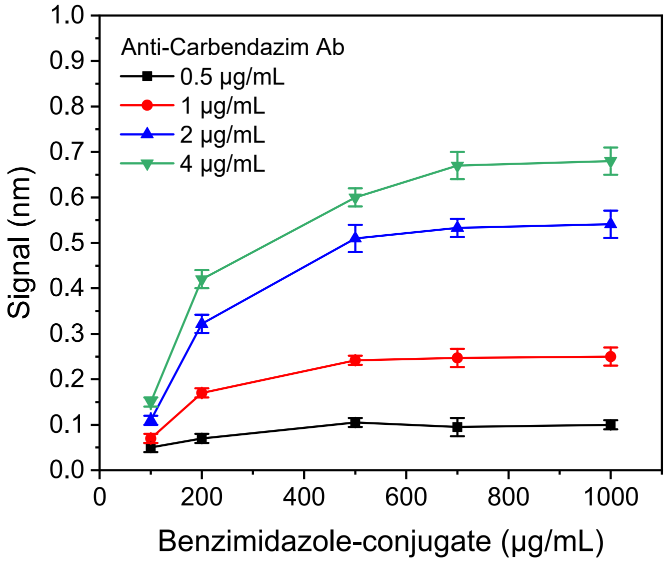

Figure S4. Signals received for zero carbendazim standard from WLRS chips coated with benzimidazole conjugate concentrations ranging from 100 to 1000 µg/mL when assayed with anti-carbendazim antibody solutions of 0.5 (black squares), 1 (red circles), 2 (blue up triangles) or 4 µg/mL (green down triangles). Each point is the mean value of measurements obtained by three chips ± SD.

ii) Selection of anti-carbendazim antibody concentration

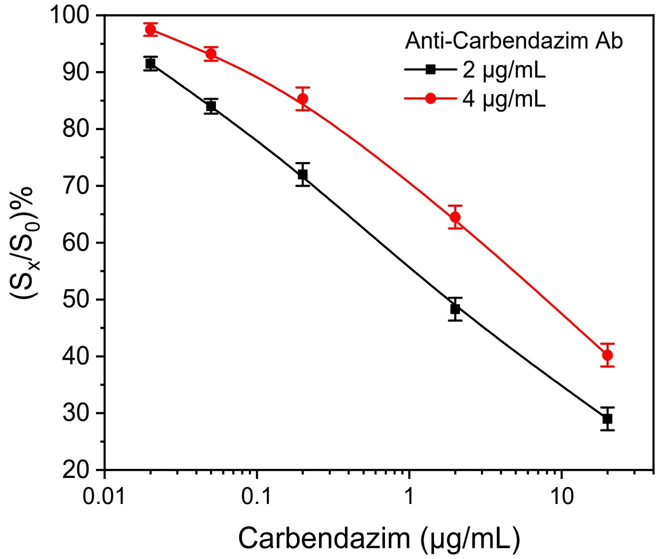

Figure S5. Calibration plots obtained from chips coated with 500 µg/mL of benzimidazole conjugate and assayed with anti-carbendazim antibody solutions with concentration 2 (black squares) or 4 µg/mL (red circles). Each point is the mean value of three measurements ± SD.
